# Effect of *β*_2_‐adrenergic receptor polymorphisms on epinephrine and exercise‐stimulated lipolysis in humans

**DOI:** 10.14814/phy2.12017

**Published:** 2014-05-20

**Authors:** Shichun Du, Michael J. Joyner, Timothy B. Curry, John H. Eisenach, Christopher P. Johnson, William G. Schrage, Michael D. Jensen

**Affiliations:** 1 Endocrine Research Unit, Mayo Clinic, Rochester, Minnesota; 2 Shanghai Jiao Tong University School of Medicine, Shanghai, China; 3 Anesthesiology Research, Mayo Clinic, Rochester, Minnesota; 4 Department of Kinesiology, University of Wisconsin, Madison, Wisconsin

**Keywords:** Body composition, indirect calorimetry, oxygen consumption, palmitate kinetics

## Abstract

The *β*_2_‐adrenergic system is an important regulator of human adipose tissue lipolysis. Polymorphisms that result in amino acid substitutions in the *β*_2_‐adrenergic receptor have been reported to alter lipolysis. We hypothesized that variations in the amino acid at position 16 of the *β*_2_‐adrenergic receptor would result in different lipolytic responses to intravenous epinephrine and exercise. 17 volunteers homozygous for glycine at position 16 (Gly/Gly, nine female) and 16 volunteers homozygous for arginine at position 16 (Arg/Arg, eight female) of the *β*_2_‐adrenergic receptor participated in this study. On one study day participants received infusions of epinephrine at submaximal (5 ng kg^−1^ min^−1^) and maximal (40 ng kg^−1^ min^−1^) lipolytic doses. The other study day volunteers bicycled for 90 min at 50–60% of maximum oxygen consumption (VO_2_max). [9,10‐^3^H] Palmitate was infused both days to measure free fatty acid – palmitate kinetics. Oxygen consumption was measured using indirect calorimetry. Palmitate release rates in response to epinephrine and exercise were not different in the Gly/Gly and Arg/Arg participants. The only statistically significant difference we observed was a lesser ΔVO_2_ in Arg/Arg volunteers in response to the submaximal epinephrine infusion. The polymorphisms resulting in Arg/Arg and Gly/Gly at position 16 of the *β*_2_‐adrenergic receptor do not result in clinically meaningful differences in lipolysis responses to epinephrine or submaximal exercise.

## Abstract (precis)

e12017

Some studies suggest the polymorphism in the *β*_2_‐adrenergic receptor have effects on fatty acid mobilization from adipose tissue. We measure lipolysis using tracer kinetics in volunteers selected for different genotypes at position 16 of the *β*_2_‐adrenergic receptor. We found that polymorphisms resulting in Arg/Arg and Gly/Gly at position 16 of the *β*_2_‐adrenergic receptor do not result in clinically meaningful differences in lipolysis responses to epinephrine or submaximal exercise.

## Introduction

Catecholamines, via their action on the *β*_2_‐adrenergic receptor, are some of the most potent regulators of human adipose tissue lipolysis. It might be expected that genetic variations in the *β*_2_‐adrenergic receptor would affect the adipose tissue lipolytic response to catecholamines and thus adipose tissue function. The two most common polymorphisms of the *β*_2_‐adrenergic receptor are located at nucleotides 46 and 79 and they result in changes in amino acid residues at 16 and 27 of the amino terminus. A change from adenine to guanine at nucleic acid 46 results in a change from arginine to glycine at amino acid 16. A change from cytosine to guanine at nucleic acid 79 results in a change from glutamine to glutamic acid at amino acid 27. To date most studies on the intermediate physiologic effects of these common *β*_2_‐adrenergic receptor polymorphisms have focused on differences at amino acid 16. This polymorphism has been studied in relation to lipolysis and obesity. Results of previous studies are not definitive as to whether carriers of these polymorphisms differ with regard to in vivo regulation of lipolysis (Galletti et al. 2004; Oomen et al. 2005; Petrone et al. 2006).

In this study, we tested whether polymorphisms that result in different amino acids at position 16 of the *β*_2_‐adrenergic receptor result in different lipolysis responses to an epinephrine infusion or to an exercise stimulus in adult humans. We measured free fatty acid (FFA, palmitate) kinetics in response to both submaximal and maximal doses of epinephrine in individuals homozygous for the gene resulting in either glycine or arginine at position 16 of the *β*_2_‐adrenergic. In the same group of volunteers we measured the lipolytic response to a 90 min bicycle exercise stimulus at 50–60% VO_2_max. We hypothesized that individuals who are homozygous for arginine at position 16 will have blunted lipolysis responses to epinephrine stimulation and/or physical exercise.

## Research Design and Methods

### Subjects

This study was approved by the Mayo Institutional Review Board. Each volunteer provided informed, written consent. Participants were recruited from a pool of subjects that had previously been genotyped for *β*_2_‐adrenergic receptor rs1042713 (single‐nucleotide polymorphisms, SNPs 16) (Bray et al. 2000). Seventeen subjects who were homozygous for glycine at position 16 (Gly/Gly, nine female) and 16 subjects who were homozygous for arginine at position 16 (Arg/Arg, eight female) were recruited for participation in these experiments. These participants were also genotyped for polymorphisms that affect the amino acid at position 27. Because of linkage disequilibrium between positions 16 and 27, all Arg16 homozygotes in our cohort were Gln27 homozygotes. Conversely, the Gly16 homozygotes varied at position 27, such that one of these individuals was the rare Gln‐Gln27, ten were Gln‐Glu27 (heterozygous), and six were Glu‐Glu27. Each volunteer underwent a graded maximum bicycle test to assess VO_2_max and established work rates for the later exercise trials. Body composition was measured before the studies using DXA (Jensen et al. 1993).

### Protocol

All volunteers had maintained a stable weight for more than 2 months before the study. Whole body, in vivo regulation of lipolysis was studied on 2 days using a palmitate tracer to measure FFA kinetics (Miles et al. 1987). On one study day epinephrine was infused at both submaximal and maximal lipolytic doses (Divertie et al. 1997) and on another day lipolysis was stimulated using an exercise challenge. To assure consistent macronutrient intake each participant consumed all meals in the Mayo Clinic Clinical Research Unit (CRU) for 3 days before the studies. The meals provided 20% of energy as protein, 40% as fat, and 40% carbohydrate. Each volunteer was admitted to the CRU the night before the studies to minimize any physiological disruption that might alter the lipolytic responses.

On both mornings an 18‐gauge intravenous catheter was placed and used for the infusions described below; a second intravenous catheter was inserted in a retrograde fashion into a contralateral hand vein (the “heated hand vein technique”) for collection of arterialized‐venous blood samples. After obtaining a baseline blood sample to be used as a blank, an infusion of [9,10‐^3^H] palmitate (~0.3 *μ*Ci/min) was started 45 min before the blood sampling for palmitate kinetic measures was started in order to assure isotopic steady state. Four arterialized blood samples were collected over a 30‐min interval for measurement of baseline FFA kinetics and plasma hormone concentrations. A 1‐h epinephrine infusion at a submaximal (5 ng kg^−1^ min^−1^) lipolytic dose was then started and blood samples were collected every 15 min for measurement of palmitate concentration and specific activity (SA), as well as plasma insulin, glucose, and catecholamine concentrations. Oxygen consumption and carbon dioxide production were measured during each study interval. After the low‐dose epinephrine was discontinued the volunteers laid quietly in bed for 90 min. During the last 30 min of this interval, four blood samples were collected at 10 min intervals for measurement of FFA kinetics and plasma hormone concentrations prior to the 60 min maximal lipolytic epinephrine (40 ng kg^−1^ min^−1^) infusion. Blood and breath samples were collected as during the low‐dose infusion. The participants were monitored for 1 h after discontinuing the high‐dose epinephrine, during which time we collected blood and breath samples. The volunteers were then provided with their weight maintenance diet and they remained in the CRU in preparation for the exercise study that was conducted the next day.

On the second study day we infused [9,10‐^3^H] palmitate in an identical fashion to the first study day beginning 45 min before the first blood sample. Four blood samples were collected over a 30‐min interval to allow measurement of baseline FFA kinetics and plasma hormone concentrations. The volunteers then performed 90 min of bicycle exercise at 50–60% VO_2_max. Blood samples, oxygen consumption, and carbon dioxide production were measured every 15 min during exercise and during 1 h of recovery.

### Analysis of samples

Plasma palmitate concentration and specific activity were measured by high‐performance liquid chromatography (HPLC) (Miles et al. 1987). Plasma glucose was measured using a glucose analyzer (Beckman Instruments, Fullerton, CA). Insulin concentrations were measured using chemiluminescent sandwich assays (Sanofi Diagnostics, Chaska, MN). Plasma catecholamines were measured using HPLC with electrochemical detection (Causon et al. 1981).

### Calculations and statistical analysis

Palmitate rate of appearance (Ra) was calculated using nonsteady state formulas (Jensen et al. 1990). For each subject, the integrated lipolytic response (increase in palmitate Ra above baseline) was calculated for each epinephrine infusion and during exercise using area under the curve (AUC) analysis. One‐way analysis of variance (ANOVA) was performed to compare genotypes with respect to the integrated lipolytic response for each epinephrine infusion and exercise. Univariate regression analyses were used to test for correlations between ΔVO_2_/Δ plasma catecholamine concentrations and systemic palmitate Ra AUC. Analysis of covariance (ANCOVA) was used to analyze systemic palmitate Ra adjusting for differences in VO_2_/catecholamine concentrations between genotype groups. The distribution of each variable was assessed and because of a skewed distribution, plasma insulin concentrations were analyzed following log transformation. All data were analyzed using JMP 9.0 (SAS Institute, Cary, NC). Two‐tailed *P* values <0.05 were considered statistically significant.

### Power calculations

In a prior study, the mean ± SD maximal lipolytic response to epinephrine (maximal palmitate rate of appearance – Ra_max_) in seven nondiabetic subjects was 23% less than in seven diabetic subjects (117 ± 50 *μ*mol kg^−1^ h^−1^ and 152 ± 29 *μ*mol kg^−1^ h^−1^; Divertie et al. 1997). Because we interpreted this as a clinically meaningful difference, we developed the power calculations to provide a sample size that would allow us to detect a similar relative difference between the two genotypes. Based on the previous finding that the standard deviation of Ra_max_ is 32 *μ*mol kg^−1^ h^−1^, a total sample size of *N *=**32 (16 per genotype) provided the statistical power (two‐tailed α = 0.05) of 87% to detect this relative difference between genotypes. We required all subjects in the current investigation to consume a controlled diet 3 days prior to the study with the goal of further reducing the interindividual variability in lipolytic response. We included the exercise study to determine whether any differences observed in response to a pharmacological stimulus would be relevant to the most common condition known to increase lipolysis.

## Results

### Subjects

Anthropometric characteristics of the study subjects are provided in Table 1. By design, there were no significant differences in age, weight, body mass index (BMI), fat mass (FM), or fat‐free mass (FFM) between the two groups.

**Table 1.** Anthropometric characteristics of the study subjects.

	Gly/Gly (*n *=**17, F = 9)	Arg/Arg (*n *=**16, F = 8)	*P* value
Age (y)	27 ± 1	26 ± 1	0.81
Weight (kg)	72.3 ± 3.5	71.4 ± 3.8	0.53
BMI (kg/m^2^)	24.0 ± 0.7	24.2 ± 0.7	0.84
FM (%)	24.4 ± 2.3	26.9 ± 2.3	0.44
FFM (kg)	57.3 ± 3.7	52.9 ± 3.7	0.40
FM (kg)	17.6 ± 1.5	18.9 ± 1.5	0.55

Values are mean ± SEM. BMI, body mass index; F, female; FM, fat mass; FFM, fat‐free mass.

### Effect of Arg/Arg versus Gly/Gly at position 16 of the *β*_2_‐adrenoceptor gene on VO_2_ and lipolysis during epinephrine day

**Figure 1.**
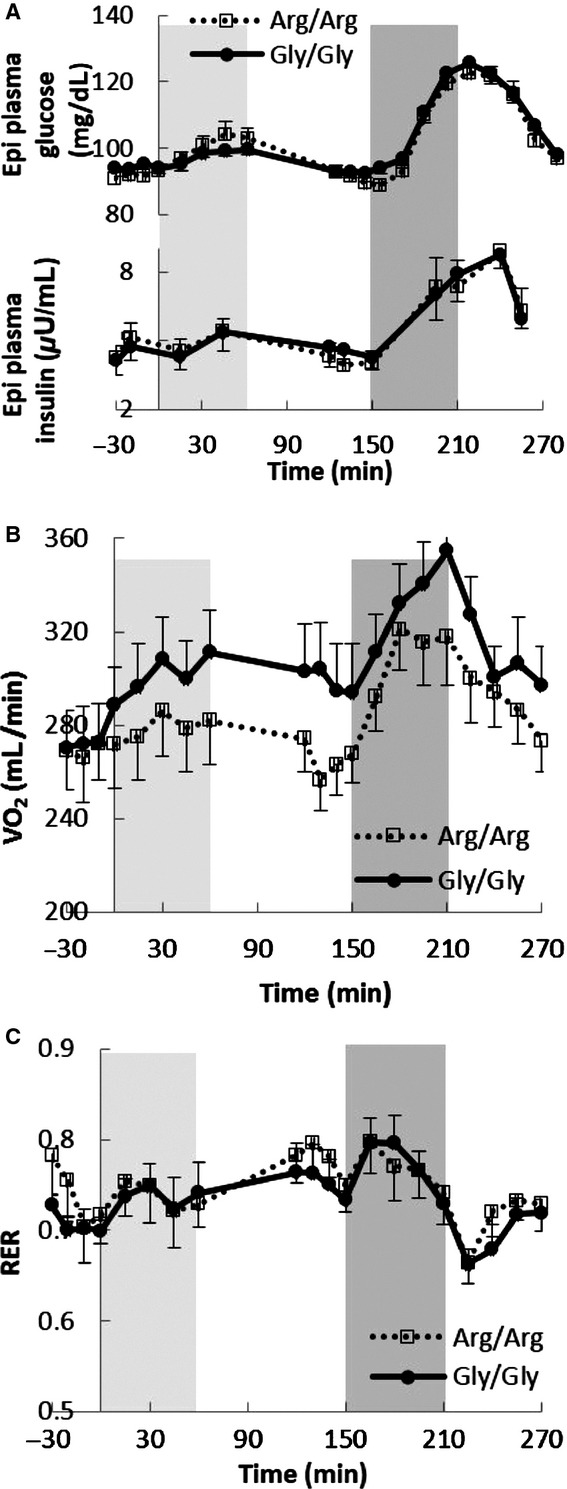
The plasma glucose and insulin concentrations (A: *n *=**14 in Gly/Gly and *n *=**15 in Arg/Arg groups), VO_2_ consumption (B: *n *=**15 in both Gly/Gly and Arg/Arg groups), and the respiratory exchange ratio (C: *n *=**15 in both Gly/Gly and Arg/Arg groups) during the epinephrine day. Column backgrounds represent low‐ (0–60 min, 5 ng kg^−1^ min^−1^) and high‐ (150–210 min, 40 ng kg^−1^ min^−1^) dose epinephrine infusion periods.

Figure 1A depicts the patterns of plasma glucose and insulin concentrations during the epinephrine infusion day. Because of difficulty collecting all blood samples, we did not have complete lipolytic responses for all volunteers; the number of participants with complete measures is provided in the tables and figures. Plasma glucose concentrations increased in both the Arg/Arg and Gly/Gly subjects in response to the low‐ (92 ± 1 to 104 ± 4 mg/dL, *P *=**0.03 and 94 ± 1 to 99 ± 1 mg/dL, *P *=**0.01, respectively) and high‐dose (91 ± 1 to 123 ± 3 mg/dL, *P *=**0.0001 and 93 ± 2 to 126 ± 4 mg/dL, *P *<**0.0001, respectively) epinephrine infusion; the changes were not different between groups. Plasma insulin concentrations were not significantly different during the low‐dose epinephrine infusion (4.7 ± 0.5 to 5.4 ± 0.5 and 4.4 ± 0.6 to 5.4 ± 0.8 *μ*U/mL, respectively, both *P *= NS) and increased to a greater degree during the high‐dose epinephrine infusion (4.1 ± 0.5 to 8.9 ± 1.1 and 4.5 ± 0.7 to 8.7 ± 0.6 *μ*U/mL, respectively, both *P *= <0.001); the responses were not significantly different between the Arg/Arg and Gly/Gly subjects.

Table 2 provides the plasma epinephrine/norepinephrine concentrations during the epinephrine infusion trial. The plasma epinephrine and norepinephrine concentrations were similar (*P *= NS) between the two groups before, during and after the epinephrine infusions.

**Table 2.** Plasma epinephrine/norepinephrine concentrations during the epinephrine infusion trial.

Epinephrine day	Gly/Gly	Arg/Arg	*P* value
Plasma epinephrine concentration (pg/mL)5			
Basal	34 ± 5	26 ± 3	0.33
Epi #1	108 ± 7	103 ± 7	0.65
Resting	33 ± 4	24 ± 4	0.39
Epi #2	543 ± 39	504 ± 37	0.91
Plasma norepinephrine concentration (pg/mL)*			
Basal	142 ± 16	128 ± 16	0.73
Epi #1	148 ± 10	153 ± 12	0.47
Resting	140 ± 14	135 ± 14	0.92
Epi #2	205 ± 21	189 ± 12	0.59

Values are mean ± SEM. Epi #1, epinephrine infusion at rate of 5 ng kg^−1^ min^−1^; Epi #2, epinephrine infusion at rate of 40 ng kg^−1^ min^−1^. There were no significant differences between Gly/Gly and Arg/Arg subjects.

* *n *=**14 in Gly/Gly and *n *=**15 in Arg/Arg groups.

Figure 1B shows oxygen consumption (VO_2_) during the epinephrine infusion study. Baseline VO_2_ was similar (*P *= NS) in the Arg/Arg and Gly/Gly subjects and increased slightly in response to the low‐ and high‐dose epinephrine infusions in both groups. The average increase during the low‐dose epinephrine infusion was less in Arg/Arg than Gly/Gly participants (Δ = 10 ± 5 vs. 26 ± 6 mL/min, *P *=**0.03). However, the average increase was similar in Arg/Arg and Gly/Gly subjects (Δ = 45 ± 8 vs. 47 ± 5 mL/min, *P *= NS) during the high‐dose epinephrine infusion. The changes in respiratory exchange ratio (RER) during the epinephrine infusion day in the two groups are depicted in Figure 1C. There were no significant between‐group differences, implying that subtrate oxidation did not differ between Arg/Arg and Gly/Gly subjects.

**Figure 2.**
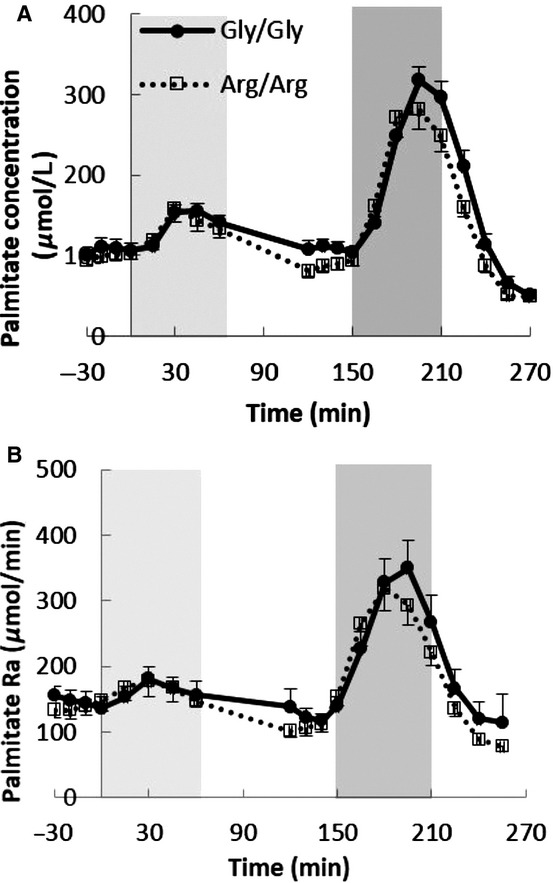
(A) The plasma palmitate concentration and (B) the rate of plasma palmitate appearance (Ra) during the epinephrine study. Column backgrounds represent low‐ (0–60 min, 5 ng kg^−1^ min^−1^) and high‐ (150–210 min, 40 ng kg^−1^ min^−1^) dose epinephrine injection periods. *n *=**16 in both Gly/Gly and Arg/Arg groups for the epinephrine study.

Figure 2A and B shows the plasma palmitate concentration and palmitate Ra responses, respectively, during the low‐ and high‐dose epinephrine infusions. The palmitate concentration and Ra increased during low‐dose epinephrine infusion and returned to preinfusion levels following discontinuation of the infusion. Baseline palmitate concentration (107 ± 9 vs. 100 ± 6 *μ*mol/L) and Ra (146 ± 15 vs.137 ± 12 *μ*mol/min, respectively) were not different between Arg/Arg and Gly/Gly subjects. During low‐ and high dose of epinephrine infusion, palmitate concentration and Ra peaked at 30 and 45 min, respectively, and decreased thereafter. As assessed using AUC analysis of palmitate concentration and Ra, the two groups had similar lipolytic responses to the low‐ (1600 ± 476 vs.1750 ± 312 *μ*mol/L per 60 min and 853 ± 540 vs.859 ± 485 *μ*mol/60 min, both *P *= NS) and high‐ (7970 ± 852 vs. 7177 ± 489 *μ*mol/L per 60 min and 8290 ± 1188 vs.8720 ± 1165 *μ*mol/60 min, both *P *= NS) epinephrine infusions.

### Effect of Arg/Arg versus Gly/Gly at position 16 of the *β*_2_‐adrenoceptor gene on VO_2_ and lipolysis during exercise

**Figure 3.**
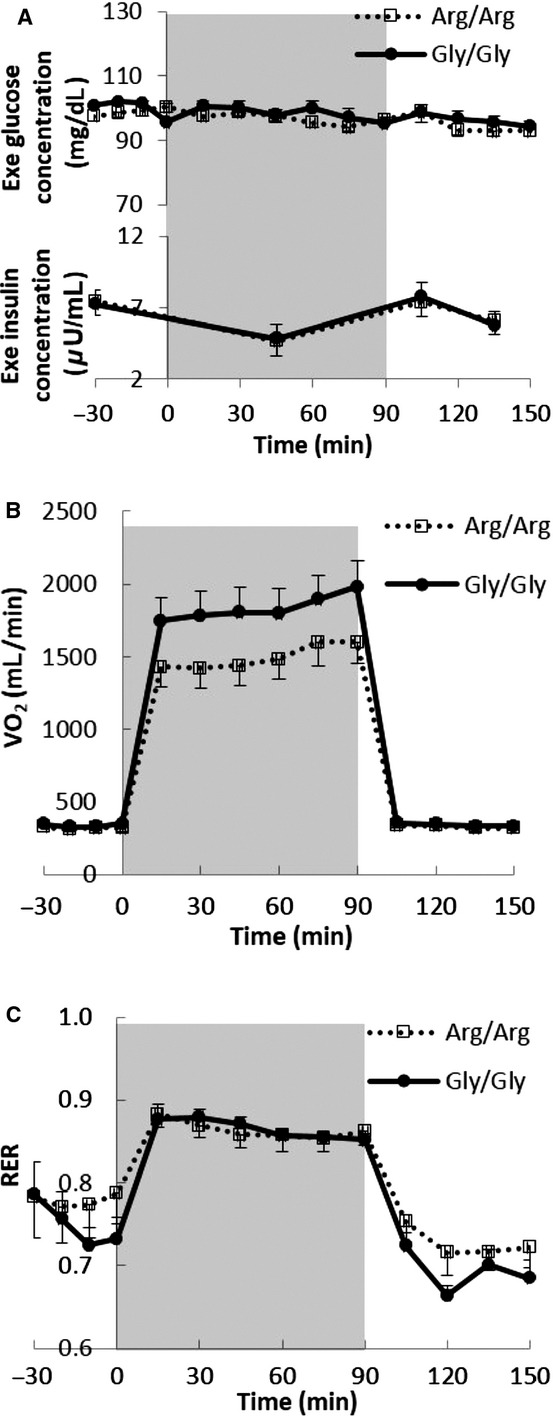
The plasma glucose and insulin concentrations (A; *n *=**15 in both Gly/Gly and Arg/Arg groups), VO_2_ volume (B: *n *=**14 in both Gly/Gly and Arg/Arg groups), and RER (C: *n *=**14 in both Gly/Gly and Arg/Arg groups) during the exercise day. Column background (0–90 min) represent exercise period.

Figure 3A shows the plasma glucose and insulin concentrations before, during, and after exercise. During exercise the plasma glucose concentrations remained stable and similar (average 97 ± 1 vs. 98 ± 1 mg/dL, respectively, *P *= NS) in Arg/Arg and Gly/Gly subjects. Plasma insulin concentrations decreased during exercise (7.5 ± 0.8 to 4.7 ± 0.7 *μ*U/mL, *P *=**0.02 and 7.3 ± 0.6 to 4.9 ± 0.6 *μ*U/mL, *P *=**0.006, respectively) in Arg/Arg and Gly/Gly subjects and returned to baseline during recovery from exercise.

Table 3 provides the plasma epinephrine/norepinephrine concentrations during the exercise study. The plasma epinephrine concentrations were similar (*P *= NS) between the two groups; plasma norepinephrine concentrations were not different during basal and recovery period but were somewhat less (*P *=**0.05 not adjusted for multiple comparisons) in Arg/Arg subjects during exercise.

**Table 3.** Plasma epinephrine/norepinephrine concentrations during the physical exercise trial.

Exercise day	Gly/Gly	Arg/Arg	*P* value
Plasma epinephrine concentration (pg/mL)*			
Basal	85 ± 7	64 ± 7	0.13
Exercise	182 ± 25	109 ± 18	0.32
Recovery	72 ± 7	63 ± 12	0.51
Plasma norepinephrine concentration (pg/mL)*			
Basal	294 ± 21	276 ± 31	0.47
Exercise	1213 ± 103	854 ± 90	0.05
Recovery	276 ± 22	257 ± 16	0.90

Values are mean ± SEM. There were no significant differences between Gly/Gly and Arg/Arg subjects.

* *n *=**15 in both Gly/Gly and Arg/Arg groups.

Figure 3B shows the VO_2_ during the exercise trial. VO_2_ was similar during basal and recovery period and slightly lower (*P *=**0.11) in Arg/Arg than Gly/Gly subjects during exercise. RER increased significantly during physical exercise (0.77 ± 0.02 to 0.86 ± 0.01, *P *=**0.003 and 0.75 ± 0.02 to 0.87 ± 0.01, *P *=**0.0003, respectively) in Arg/Arg and Gly/Gly subjects, consistent with greater proportionate carbohydrate oxidation at this level of exercise intensity (Fig. 3C).

**Figure 4.**
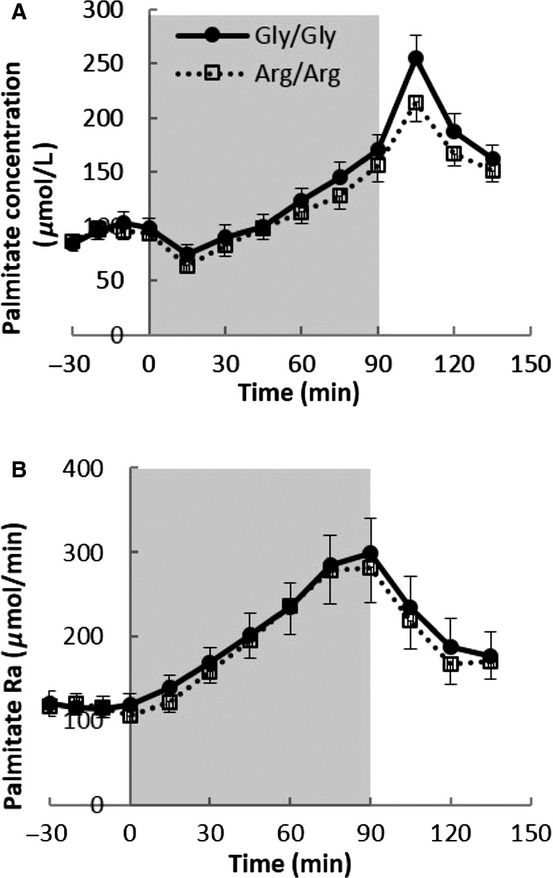
(A) The plasma palmitate concentration and (B) the rate of plasma palmitate appearance (Ra) during the exercise study. Column background (0–90 min) represent exercise period. *n *=**16 in both Gly/Gly and Arg/Arg groups for the exercise study.

Figure 4A and B show the plasma palmitate concentration and Ra responses to 90 min of bicycle exercise. The patterns of plasma palmitate concentrations were not different between the two groups before, during, or after exercise. Baseline palmitate concentration/Ra (93 ± 8 vs. 95 ± 8 *μ*mol/L and 115 ± 11 vs. 117 ± 13 *μ*mol/min, both *P *= NS) were not different between Arg/Arg and Gly/Gly subjects. When compared by the AUC above baseline analysis of palmitate Ra, the two groups had similar lipolysis response to 90‐min physical exercises (7642 ± 1504 vs. 7997 ± 1262 *μ*mol/90 min, *P *= NS).

### Relationship between VO_2_/plasma catecholamine concentrations and palmitate Ra

**Figure 5.**
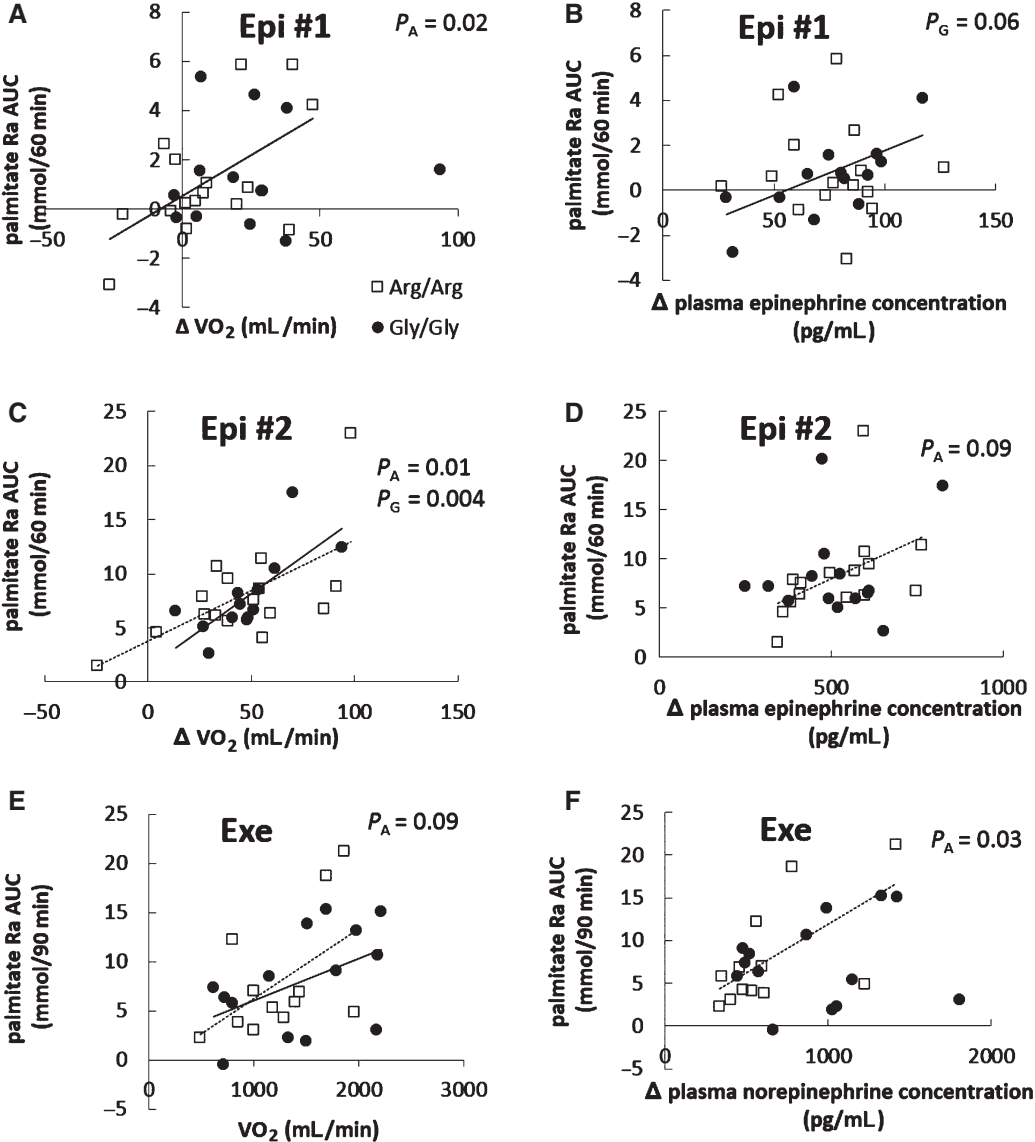
Relationship between ΔVO_2_ and palmitate Ra AUC in submaximal (A: *n *=**16 in Arg/Arg and *n *=**12 in Gly/Gly group), maximal (C: *n *=**16 in Arg/Arg and *n *=**13 in Gly/Gly group) epinephrine infusion, and exercise trial (E: *n *=**12 in Arg/Arg and *n *=**14 in Gly/Gly group). Relationship between plasma catecholamine concentrations and palmitate Ra AUC in submaximal (B: *n *=**15 in Arg/Arg and *n *=**14 in Gly/Gly group), maximal (D: *n *=**15 in Arg/Arg and *n *=**13 in Gly/Gly group) epinephrine infusion, and exercise trial (F: *n *=**12 in Arg/Arg and *n *=**14 in Gly/Gly group) in Arg/Arg (□) and Gly/Gly (●) subjects. Regression lines were depicted where there were statistically significant or borderline differences in Arg/Arg (dashed line, *P*_A_) and Gly/Gly (solid line, *P*_G_) subjects.

Figure 5 shows the relationship between changes in plasma epinephrine, plasma norepinephrine, and VO_2_ as they relate to changes in palmitate Ra. There was a positive correlation (*P *=**0.02) between the palmitate Ra AUC and ΔVO_2_ during the submaximal epinephrine infusion in Arg/Arg subjects (Fig. 5A). There were also significant, positive correlations between the palmitate Ra AUC and ΔVO_2_ during the maximal epinephrine infusion in both Arg/Arg subjects and Gly/Gly subjects (Fig. 5C). The correlation between palmitate Ra AUC and plasma epinephrine concentrations during the submaximal and maximal epinephrine infusions were borderline (*P *=**0.06) in Arg/Arg and Gly/Gly (*P *=**0.09) volunteers, respectively (Fig. 5B and D). The associations between ΔVO_2_ and palmitate Ra AUC and plasma norepinephrine and palmitate Ra AUC during exercise are depicted in Figure 5E and F (plasma epinephrine concentrations did not correlate with exercise palmitate Ra AUC). There were not significant differences in these relationships between the Arg/Arg and Gly/Gly participants during exercise. Adjusting for ΔVO_2_ and Δ plasma epinephrine (epinephrine day) or Δ plasma norepinephrine concentration (exercise day) did not uncover differences in the palmitate Ra AUC between the two groups.

## Discussion

These studies were designed to determine whether genetic variations in the *β*_2_‐adrenergic receptor result in clinically meaningful variations in effective adipose tissue lipolysis responses to pharmacological and physiological stimuli. A receptor polymorphism that results in blunted lipolysis would have important implications for the regulation of lipid metabolism in humans and might have important long‐term implication of body composition. Contrary to our hypothesis, the lipolytic responses to epinephrine and physical exercise were not different in individuals homozygous for arginine or glycine at position 16 of the *β*_2_‐adrenergic receptor, although Arg/Arg subjects had lower ΔVO_2_ during the submaximal epinephrine infusion.

Previous studies using in vitro experimental systems showed the Gly16 allele resulted in significant differences in agonist promoted downregulation of the *β*_2_‐adrenergic receptor without a change in agonist binding or functional coupling to intracellular signaling proteins (Green et al. 1994). However, in vitro differences in lipolysis may not translate readily to the in vivo situation because of the complicated regulation of neuroendocrine and adipose tissue blood flow (Clutter et al. 1980; Hjemdahl and Linde 1983). Our data suggest that these in vitro differences in function cannot be readily extrapolated to in vivo regulation of FFA release.

The results of recent studies on whether *β*_2_‐adrenergic receptor polymorphisms alter adipose tissue metabolism have been inconsistent. Galletti et al. (2004) found no differences in body mass index, serum triglycerides concentrations, or HOMA index between middle‐age men with the Arg/Arg and Gly/Gly variations and likewise Petrone et al. (2006) found similar serum triglyceride and LDL‐cholesterol concentrations in overweight adults with the Arg/Arg and Gly/Gly variations at codon 16 of the *β*_2_‐adrenergic receptor. In contrast, Eriksson et al. (2004) reported that cultured human subcutaneous fat cells with Arg16Arg–Gln27Gln haplotype had reduced catecholamine‐induced lipolysis. Large et al. (1997) found a fivefold greater agonist *β*_2_‐adrenergic receptor affinity in adipocytes from Gly/Gly compared with Arg/Arg subjects, although codon 16 was not associated with variation in obesity variables. Finally, Meirhaeghe et al. (2000, 2001) reported that Arg/Arg women had lower plasma FFA and greater FFA suppression after glucose load and the effect of Arg/Arg genotype on the suppression of NEFA was modified by physical activities. However, plasma FFA concentrations are an imperfect indicator of FFA kinetics. Women have greater lipolysis rates than men at comparable FFA concentrations (Nielsen et al. 2003) and the divergence of FFA concentrations and Ra (flux) during exercise (Fig. 4A and B), which has been previously reported (Havel et al. 1964; Ahlborg et al. 1974), highlights the need for using FFA tracers to understand human lipolysis in vivo.

Some limitations of previous studies are the difficulty knowing whether measurements of the lipolysis from cultured adipocytes translate into in vivo responses and the measurement of plasma FFA and glycerol concentrations rather than kinetics. To the best of our knowledge, this is the first study to investigate the association of *β*_2_‐adrenergic receptor polymorphisms that result in amino acid substitutions at position 16 result in in vivo differences in FFA kinetics. The two groups were similar to within ≤5% for all measures of palmitate release rates. This is well within the day‐to‐day variation in intraindividual postabsorptive FFA flux (Nielsen et al. 2003).

We found Arg/Arg subjects had a lesser increase in VO_2_ in response to the lower dose epinephrine infusion, similar to the findings of Oomen et al. (2005), who measured the increase in VO_2_ in Gly/Gly and Arg/Arg subjects in response to salbutamol. We did not find statistically significant differences in the VO_2_ increments between Gly/Gly and Arg/Arg subjects in response to the higher dose epinephrine infusion or bicycle exercise. Adipose tissue is likely not responsible for the thermic response to epinephrine (Jensen et al. 1996), which may explain why it is possible to observe divergent lipolytic and thermogenic responses in adults who differ with respect to the *β*_2_‐adrenergic receptor amino acid content. During physical exercise, skeletal muscle plays the predominant role in energy metabolism and utilizes a large amount of glucose for oxidative metabolism (Fig. 3C, RER elevated above baseline), so the significance of *β*_2_‐adrenergic receptor may be expected to be limited.

The study population was recruited based on homozygous position 16, and we acknowledge that some investigations have reported on the physiological importance of position 27 (Eriksson et al. 2004 #3210). As noted, all Arg16 homozygotes in our cohort were Gln27 homozygotes, whereas the Gly16 homozygotes varied at position 27. The smaller numbers of individuals with the Gln‐Gln27, Gln‐Glu27, and Glu‐Glu27 polymorphisms left us with insufficient statistical power to detect an interaction effect among position 16 + 27 haplotypes. When comparing position 27 irrespective of position 16 (Gln27 homozygotes, *n *=**17; Glu27 homozygotes, *n *=**6), we found no evidence to suggest that position 27 was associated with differences in the lipolysis responses to epinephrine or submaximal exercise (data not shown).

Some limitations to our results must be acknowledged. First, epinephrine is a nonselective agonists of α and *β* (*β*_1_, *β*_2_, and *β*_3_) adrenergic receptors. Thus, the responses we observed are not specific to the *β*_2_‐adrenergic receptor, although they are representative of two of the common physiological stimulants of lipolysis, stress (epinephrine) and physical activity. Another limitation is that all of our volunteers were Caucasian, and thus we cannot extrapolate these results to other racial groups.

In summary, heritable differences in the *β*_2_‐adrenergic receptor at position 16 appear to be inconsequential with respect to lipolysis in vivo. Our hypothesis that individuals who are homozygous for arginine at position 16 of the *β*_2_‐adrenergic receptor have blunted lipolysis was not supported in this study, although a lesser thermic response to low‐dose epinephrine was observed in participants with Arg/Arg genotype.

## Acknowledgment

The authors are grateful to the participants for playing an integral role in making this research possible. In addition, the authors thank the Mayo Clinic CRU staff members.

## Conflict of Interest

None declared.
